# Minimal important differences and response shift in health-related quality of life; a longitudinal study in patients with multiple myeloma

**DOI:** 10.1186/1477-7525-8-79

**Published:** 2010-08-03

**Authors:** Ann K Kvam, Finn Wisløff, Peter M Fayers

**Affiliations:** 1 Dept. of Haematology, Oslo University Hospital, Ullevaal, Norway; 2 Faculty of Medicine, University of Oslo, Oslo, Norway; 3 Division of Applied Health Sciences, University of Aberdeen, Aberdeen, Scotland; 4 Pain and Palliation Research Group, Dept. of Cancer Research and Molecular Medicine, Faculty of Medicine, Norwegian University of Science and Technology (NTNU), Trondheim, Norway

## Abstract

**Background:**

We previously reported that changes of 6-17 percent in the EORTC QLQ-C30 scores are regarded important by patients with multiple myeloma and thus may be considered as Minimal Important Differences (MIDs). However, patients' internal standard of measurement may have changed over time (response shift, RS). In the present work, we evaluated whether myeloma patients experience RS and if this could affect the MID-estimates.

**Methods:**

Between 2006 and 2008, 239 patients with multiple myeloma completed the EORTC QLQ-C30 at baseline (T1) and after three months (T2). At T2, patients were asked if they had noticed any change in the domains pain, fatigue, physical function and global quality of life. They were also asked to give a retrospective judgment of their baseline values on all the four domains.

**Results:**

We found clear evidence of RS in myeloma patients. However, there were differences in both magnitude and direction between patients who stated that they improved and those who deteriorated. Deteriorating patients retrospectively reported better health-related quality of life at T1 for the domains pain, fatigue and physical function. In these patients, MIDs adjusted for RS were observed to increase up to 12 percentage points. In contrast, for patients stating that they improved, we only found evidence of statistically significant RS in the domain global quality of life.

**Conclusions:**

MIDs estimated from pre-test/post-test data appeared to be robust against RS in patients reporting improvement over 3-months. This could indicate that RS has a minimal impact on the results in patients who respond to treatment, and that RS may not have an important impact on interpretation of changes reported in clinical trials where an improvement occurs.

Although the effect sizes of the RSs were small, RS in deteriorating patients may have an important impact on the interpretation of changes reported in clinical trials.

**Trial registration:**

The study is registered at clinicaltrials.gov, identifier NCT00290095.

## Background

A challenge in the interpretation of health-related quality of life (HRQOL) data in clinical research is that HRQOL is self-reported by the patient, and might be influenced by psychological phenomena such as adaptation to illness. Patients who experience changes in health often accommodate and adapt to these changed conditions. When measuring changes in HRQOL with a pre-test (assessment prior to intervention)/post-test (assessment after intervention) design, as in a Randomized Controlled Trial (RCT), adaption to increased symptom level or impaired HRQOL can affect results, a change referred to as *response shift *(RS) [1]. Sprangers and Schwartz defined RS in the field of HRQOL as a change in the meaning of an individual's self-reported HRQOL [2]. It can be divided into 1) *Reconceptualization *(i.e. a re-definition of HRQOL), 2) *Reprioritization *(i.e. a change in the importance attributed to component domains constituting HRQOL) and 3) *Recalibration *(i.e. a change in a patient's internal standards of measurements). The most widely used approach for assessing changes in a patient's internal standard is the retrospective pre-test design (then-test) [1,3]. At post-test, patients are retrospectively asked to provide a renewed judgment of their HRQOL at baseline (pre-test). The then-test is ideally completed simultaneously with or in close proximity to the post-test, assuming patients rate their HRQOL on both tests using the same internal standards.

During the last years, several studies have found evidence for the occurrence of RS in HRQOL in cancer patients -e.g. [4-10]. RS may sometimes be the result of an adaptive response to a changed health status, and may then be viewed as a positive phenomenon to patients. However, the altered meaning of HRQOL over time poses a challenge to clinicians in the interpretation of changes in HRQOL. In a study by Visser et al, fatigue was assessed in 216 cancer patients before and after treatment with radiotherapy [4]. When the conventional pre-test was compared to the post-test, no differences in fatigue were found. This might lead to the conclusion that radiotherapy does not affect fatigue. However, when the then-test was used as the measure of fatigue at baseline, there appeared to be a statistically significant increase in fatigue after treatment.

The magnitude and importance of the RS phenomenon remains unsolved. A meta-analysis by Schwartz et al suggested that RS may play a significant role in HRQOL research and that the direction of this shift varies across studies [11]. In a previous report we attempted to determine the clinical significance of changes in quality-of-life scores in patients with multiple myeloma (MM) [12]. MM is an incurable malignant disease of the bone marrow with an expected median survival of five years [13]. At diagnosis, myeloma patients report a pronounced impairment of HRQOL, with reduced physical functioning, fatigue and pain as the major problems [14]. Aims of treatment are to control disease, maximize quality of life and prolong survival. Hence, HRQOL is an important outcome in clinical trials. We estimated the Minimal Important Difference (MID) in patients with MM for the HRQOL instrument, the EORTC QLQ-C30. MID is defined as "the smallest difference in score in the domain of interest which patients perceive as beneficial and which would mandate, in the absence of troublesome side effects and excessive cost, a change in the patients' management" [15]. Our results suggested that a change in the EORTC QLQ-C30 score in the range of approximately 6-17 (on a 0-100 scale) is considered important by patients with MM. Here, we evaluate whether patients experienced RS, and if so its magnitude and direction. We also explore how RS affects the MID-results and whether RS impacts on the interpretation of HRQOL results in clinical trials.

## Methods

### Patients

Patients with MM, irrespective of their disease status (newly diagnosed, plateau phase, relapsed) or treatment, were enrolled from January 2006 to April 2008. Eligibility was expected survival greater than three months and ability to complete a self-report questionnaire in Norwegian. Consecutive patients admitted to 17 hospitals in the South-Eastern Norway Regional-Health-Authority, a region representing about 50% of the Norwegian population, were recruited. Written informed consent was obtained from all participants. The Helsinki Declaration guidelines were followed. The Regional Committee for Medical Research Ethics, Health region I, Norway, approved the study.

### Questionnaire

HRQOL was measured using the EORTC QLQ-C30, a cancer-specific questionnaire with 30 items [16]. The questionnaire is composed of five functional scales, three symptom scales, a global health/quality of life scale, and six single items. All scores were calculated and transformed to a 0-100 scale according to EORTC methods [17]. For the functional scales and global health status, higher scores represent a higher level of functioning. In the symptom scales and single items, higher scores represent more symptoms or difficulties. The questionnaire is reliable and valid for MM patients [18].

### Interview and Then-test approach

Patients completed the EORTC QLQ-C30 at inclusion (T1) and after three months (± 2 weeks window) (T2). At T2, a structured interview was performed and the patients were asked: "Compared with the last time you filled in the questionnaire (T1, date mentioned to the patients), has your quality-of-life improved, stayed the same or deteriorated?" The response choices ranged on a seven-point scale from 1 = much better to 7 = much worse. This global rating of change (GRC) question was asked for the four domains physical functioning, fatigue, pain and global quality of life. Because of small sample sizes in some of the GRC categories, we pooled the data into three categories (improved, unchanged, deteriorated) to yield sufficient numbers of cases in each category. "Improved" included much better, moderately better and a little better and "deteriorated" included a little worse, moderately worse and much worse for the four domains. MIDs for *improvement *and *deterioration *were defined as the mean score changes in these domains for patients declaring improvement or deterioration. During the article we would use *improved *as shorthand for patients "who reported themselves as improved", and similarly for *deteriorated *and *unchanged *patients.

After the GRC questions, the patients were asked to provide a renewed judgment of their baseline ratings of the EORTC QLQ-C30 for the four domains (Then-test). The questions were asked in past tense for each of the 12 items included in these domains. We emphasized that the purpose of the then-test was not to recall their previous answers but to provide a renewed judgment of their HRQOL at baseline.

The mean difference between the pre-test and then-test scores was used to provide an estimate of the direction and magnitude of the RS effect. Observed changes were calculated by the difference between the mean post-test and pre-test scores while adjusted changes were measured as the difference between mean post-test and then-test scores.

### Statistical methods

Wilcoxon tests for pair differences were used to calculate the significance of differences between pre-test, post-test and then-test. We divided the patients into groups according to whether they thought they were *improved, unchanged *or *deteriorated *for the four domains.

To examine the magnitude of recalibration RS, effect sizes (ES) were calculated by dividing the mean score changes by the standard deviation at baseline (T1). We used Cohen's generally accepted criteria for interpreting the magnitude of an ES: > 0.20 is a small change, > 0.50 a moderate change, and > 0.80 a large change [19].

The GRC results and the observed and adjusted changes all appeared approximately to reflect underlying normal distributions. Analysis of variance (ANOVA) was performed and F-statistics values were calculated to see which approach (a seven-point GRC scale, observed changes or adjusted changes) was most efficient at detecting changes in phases of the disease (newly diagnosed, relapse/progression or stable disease). Newly diagnosed patients were expected to improve, relapsed patients to deteriorate and patients with a stable disease to stay unchanged. The relative efficiency of a test is measured by the ratio of the F-statistics values [20]

#### Missing data

If any item was missing in the first questionnaire (T1), we accepted the data as missing. For the second questionnaire (T2), the forms were checked and if any item was missing the patients were asked to fill it in before the interview. Still, if any of the constituent items in a scale were missing, the scale score for that patient was excluded from the statistical analyses.

#### Sample size calculation

The study primarily aimed to estimate the MID and sample size calculation was based on being able to detect a MID of 0.50 × SD, yielding a sample size of 260 patients. The response shift evaluation is descriptive and so the impact of sample size is indicated by confidence intervals around the estimates.

The statistical analysis was performed using The Statistical Package for the Social Sciences (SPSS), version 16 (SPSS Inc., Chicago, IL, USA).

## Results

### Study sample

260 patients were recruited, and 239 (92%) who filled in both questionnaires were interviewed. Of the 21 patients lost to follow up, seven had died and nine were too ill to complete the questionnaire at T2. For the remaining five cases, the reason for lack of follow-up was administrative problems. Table 1 shows patients' characteristics. Fifty-seven percent of the patients completed the post-test and the then-test within the same or next day while 99% completed them within a week (range 0-22 days). At baseline, 0.6% of the items were missing from the EORTC QLQ-C30 questionnaires, which decreased to 0.3% at follow-up. Missing items appeared randomly distributed across domains.

**Table 1:** Patient characteristics

	Multiple myeloma patients N = 239
Age (year)	
Median (range)	66 (36-89)
Sex, no. (%)	
Male	128 (54)
Female	111 (46)
Phase of disease, no. (%)	
Newly diagnosed	87 (36)
Stable disease	80 (34)
Relapse/progression	69 (29)
Multiple myeloma treatment during the study, no (%)	
No treatment	86 (36)
MP +/- Thalidomide	55 (23)
ASCT, newly diagnosed	33 (14)
ASCT, relapse	8 (3)
Velcade	7 (3)
Thalidomide	32 (13)
Other	15 (6)
Unknown	3 (1)

MP: Melphalan and Prednisone

ASCT: Autologous stem cell transplantation

### EORTC QLQ-C30 mean scores

Overall, for patients who *improved*, the EORTC QLQ-C30 at post-test showed statistically significant (p < 0.01) better scores (less symptoms and higher functioning) than at pre-test and then-test (Table 2). For patients who *deteriorated*, the EORTC QLQ-C30 at post-test showed consistently worse scores (more symptoms and lower functioning) than at pre-test and then-test (p = 0.01). There were no significant changes in the EORTC QLQ-C30 score from pre-test to post-test for the *unchanged *patients. However, for pain and fatigue, patients reporting no change had statistically significant more symptoms at post-test than at then-test (p < 0.01).

**Table 2:** EORTC QLQ-C30 scores for the pre-, post- and then-test

	n	Pre-test (T1), mean (SD)	**p**^**α**^	Post-test (T2), mean (SD)	**p**^**β**^	**Then-test**, mean (SD)
Pain*						
*improved*	58	52.6 (32.7)	< 0.01	37.9 (27.5)	< 0.01	55.5 (24.5)
*unchanged*	126	28.7 (27.8)	0.29	27.6 (28.0)	< 0.01	22.5 (26.1)
*deteriorated*	50	46.0 (27.7)	< 0.01	63.3 (28.6)	< 0.01	36.3 (31.0)
Fatigue*						
*improved*	56	51.0 (22.9)	< 0.01	37.5 (21.4)	< 0.01	51.4 (23.3)
*unchanged *	119	40.4 (24.6)	0.17	38.7 (26.1)	< 0.01	34.2 (25.4)
*deteriorated*	58	56.3 (25.3)	0.01	64.0 (23.7)	< 0.01	43.5 (25.3)
**Physical function^†^**						
*improved*	73	59.9 (22.3)	< 0.01	66.3 (20.4)	< 0.01	57.5 (24.2)
*unchanged *	96	69.7 (21.9)	0.77	70.4 (21.5)	0.15	71.7 (22.7)
*deteriorated*	58	60.8 (22.6)	< 0.01	48.1 (21.2)	< 0.01	68.3 (21.9)
**Quality of life^†^**						
*improved*	79	59.0 (26.1)	< 0.01	66.6 (21.0)	< 0.01	51.9 (19.5)
*unchanged *	110	64.2 (22.1)	0.69	64.5 (21.5)	0.44	63.2 (20.1)
*deteriorated*	49	48.6 (20.9)	< 0.01	36.6 (20.2)	< 0.01	48.8 (16.0)

*For the symptom scales pain and fatigue, higher scores represent more symptoms or difficulties

^† ^For the physical function and global quality of life scales, higher scores mean better functioning

SD: Standard deviation

p^α ^-values denote differences between scale-means at T1 and T2

p^β ^-values denote differences between scale-means at T2 and then-test

### Magnitude and direction of RS

**Figure 1:**
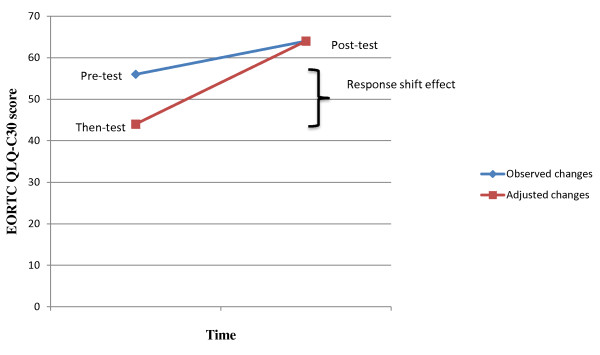
**Observed and adjusted scores of the EORTC QLQ-C30 for MM patients *deteriorated *in fatigue (n = 58)**. The patients evaluated their fatigue in retrospect as better (less fatigue) than they did at T1. The difference between the pre-test and then-test score is the response shift effect.

Table 3 summarizes the magnitude and direction of RS (pre-test - then-test scores). Overall, there were differences in both the magnitude and direction of RS between patients who *improved *and those who *deteriorated*. Patients *improving *from T1 to T2 retrospectively underestimated their baseline values on all the four dimensions. However, a statistically significant score difference (p < 0.01) emerged only for the global quality of life dimension. In contrast, among patients who *deteriorated*, the participants retrospectively overestimated their baseline values on the four dimensions. Thus, there was a statistically significant RS for the domains pain, fatigue and physical function (p < 0.01). An illustration of the RS-effect for MM patients who deteriorated in fatigue is presented in Fig. 1. In the unchanged group, there was a statistically significant RS for the domains pain and fatigue (p < 0.01). In these domains, the unchanged patients retrospectively overestimated their baseline values.

**Table 3:** Magnitude and direction of response shift for the entire sample

EORTC QLQ-C30 domain	N	n	Response shift	95% CI for response shift	p-value	ES
**Pain**	**235**		**4.8**		**< 0.01**	**0.16**
*improved*		58	-2.9	-11, 5	0.49	-0.09
*unchanged*		127	6.4	3, 10	< 0.01	0.23
*deteriorated*		50	9.7	3, 17	< 0.01	0.35
**Fatigue**	**236**		**6.2**		**< 0.01**	**0.25**
*improved*		56	-0.4	-6, 5	0.82	-0.02
*unchanged*		121	6.2	3, 9	< 0.01	0.25
*deteriorated*		59	12.4	7, 18	< 0.01	0.49
**Physical function**	**230**		**-1.5**		**0.10**	**-0.07**
*improved*		74	2.9	0, 6	0.09	0.13
*unchanged*		98	-1.4	-4, 1	0.22	-0.06
*deteriorated*		58	-7.4	-12, -3	< 0.01	-0.33
**Quality of life**	**238**		**2.7**		**< 0.01**	**0.11**
*improved*		79	7.1	3, 12	< 0.01	0.27
*unchanged*		110	0.8	-2, 4	0.55	0.04
*deteriorated*		49	-0.2	-5, 4	0.86	-0.01

CI: Confidence interval

ES: Effect size (mean score change/SD_pretest_)

Response shift: Pre-test - Then-test

ESs were largest for those who *deteriorated *in the domains fatigue, pain and physical function (ESs were 0.49, 0.35 and 0.33, respectively, Table 3). Using Cohen's criteria, all of these ESs could be considered small. There were trivial ESs for the domains fatigue, pain and physical function for patients who *improved*.

### Effect on MID estimates

Table 4 shows the observed (post-test - pre-test) and adjusted (post-test - then-test) mean score changes in the EORTC QLQ-C30 for the four domains. The observed changes are defined as MIDs because patients regard these changes as a definite improvement or deterioration. MIDs (absolute values) for patients rating themselves as *improved *ranged from 6.2 (physical function) to 14.7 (pain). Patients reporting *deterioration *had MIDs (absolute values) in the range of 8.6 (fatigue) to 17.3 (pain). However, there was considerable variation in the observed scores, as shown by the wide confidence intervals. By using the adjusted, mean changes as MIDs, the EORTC QLQ-C30 scores varied from 9.3 to 17.5 for *improved *patients. Patients who *deteriorated *had adjusted mean change scores in the range of 12.2 to 27.

**Table 4:** Minimal important differences calculated by observed- and adjusted mean score changes

		Observed changes^†^ (Post-test - Pre-test)			Adjusted changes (Post-test - Then-test)		
	**N**	**Mean change**	**SD**	**95% CI**	**Mean change**	**SD**	**95% CI**

**Pain^a^**	234*		30.8*				
*improved*	58	-14.7	35.9	(-24,-5)	-17.5	27.1	(-25,-10)
*unchanged*	126	-1.7	20.9	(-5,2)	4.9	19.5	(2,8)
*deteriorated*	50	+17.3	23.1	(11,24)	+27.0	29.9	(19,35)
**Fatigue^a^**	233*		25.3*				
*improved*	56	-13.5	24.7	(-20,-7)	-13.9	26.3	(-21,-7)
*unchanged*	119	-2.0	17.3	(-5,1)	4.6	16.5	(2,8)
*deteriorated*	58	+8.6	23.4	(3,15)	+20.5	22.3	(15,26)
**Physical Function^b^**	227*		22.6*				
*improved*	73	+6.2	15.3	(3,10)	+9.3	16.1	(6, 13)
*unchanged*	96	-0.1	12.6	(-3,3)	-1.6	10.4	(4,0)
*deteriorated*	58	-12.8	19.2	(-18,-8)	-20.1	18.9	(-15,-25)
**Quality of Life^b^**	238*		23.9*				
*improved*	79	+7.6	23.7	(2,13)	+14.7	19.8	(10,19)
*unchanged*	110	0.4	19.1	(-3,4)	1.3	15.7	(-2,4)
*deteriorated*	49	-12.1	21.2	(-18,-6)	-12.2	14.7	(-8,-12)

^a^: Positive scores mean worse symptoms

^b^: Positive scores mean better functioning

†: Observed changes are defined as minimal important differences because these are the changes that patients regard as a definite improvement or deterioration)

*: Total sample (improved, no change and deteriorated)

CI: Confidence Interval

### Efficiency in detecting changes

Phase of disease was classified as newly diagnosed, stable or relapse/progression. Impact of phase of disease on GRC, observed and adjusted mean scores was explored using F-statistics values from ANOVA. There were statistically significant differences at the p < 0.05 level with the largest F-statistics value for GRC, closely followed by adjusted changes (Table 5). For pain, fatigue and physical function the GRC and adjusted changes have a relative efficiency (ratio between F statistics) of approximately three compared to the observed changes, and for global quality of life the relative efficiency is two.

**Table 5:** Results of the F-statistics from ANOVA for the domains pain, fatigue, physical function and global quality of life, by phase of disease

	Pain	Fatigue	Physical Function	Global quality of life
**GRC**	12.7*	8.0*	10.0*	11.9*
**Observed changes **(Post-test - Pre-test)	3.6*	1.6^†^	5.6*	6.0*
**Adjusted changes **(Post-test - Then-test)	10.7*	4.7*	17.0*	9.9*

GRC: global rating of change

*p < 0.05

†p = 0.2

## Discussion

The results of the present study indicate that RS exists in MM patients, mainly in those who *deteriorated *over the 3-month observation period. We found that patients who deteriorated in the domains pain, fatigue and physical function, retrospectively minimized their troubles at baseline. These changes in internalized standards could be a desirable adaptation mechanism to patients with cancer to maintain equilibrium in HRQOL in the face of loss.

Our findings are generally consistent with those of previous studies among other categories of cancer patients with deteriorating health conditions [4,5,21]. Jansen et al assessed RS in 46 patients with breast-cancer undergoing radiotherapy. They found that patients, who had deteriorated, retrospectively reported fewer symptoms at baseline. They concluded that RS measured by the then-test was stronger for deterioration in HRQOL than for improvement in HRQOL.

For patients who *improved*, there was no statistically significant evidence of RS except for the domain global quality of life. In RCTs in newly diagnosed patients with MM or cancer in general, patients are usually followed from the start of treatment and the majority of patients are expected to improve [22,23]. Thus, the RS phenomenon may arguably be disregarded in the interpretation of the HRQOL results from such trials. Our results are in contrast to findings in studies regarding patients with non-fatal disorders, where *improved *patients retrospectively have reported significantly higher disability [24,25]. Razmjou et al discussed this issue in a study of patients with total knee arthroplasty and concluded that "it appears that patients who wish to maintain a stable HRQOL would consciously or unconsciously magnify their treatment effect by endorsing a higher disability level retrospectively" [25].

We found some evidence for RS even in patients who were *unchanged *from T1 to T2, mainly for the domains pain and fatigue. On the average, these patients retrospectively underestimated their symptoms. A meta-analysis by Hagedoorn et al [7] concluded that RS is a common and significant phenomenon in HRQOL measurement, and that in cancer studies, patients with a declining HRQOL may report no decrease in their HRQOL due to positive adaptation. This could be an explanation for the findings for the unchanged group in our study.

ESs can be calculated to evaluate the importance of the observed RS. In our study, we found that the ESs of the RS were small according to Cohen's criteria with the largest ES detected for fatigue. Fatigue has been identified by patients with cancer as a major obstacle to normal functioning and a good quality of life [26]. Previous studies have suggested that fatigue is a symptom that is especially RS prone [4,21].

It is important to know the clinical significance of changes in HRQOL scores for the interpretation of the results from clinical trials. We have previously reported that a difference of 6-17 points (scale range 0-100) in the EORTC QLQ-C30 score represents a clinically meaningful change in patients with MM. In the present study, we found that by controlling for RS in patients who *improved*, the same interval for MIDs could be used. However, if we adjust for RS in patients who *deteriorated*, larger MIDs (12-27 points) are obtained. The question is still: does adjusting for RS provides more reliable estimates of MIDs?

The F-statistics values from ANOVA indicates that the GRC is the most effective method for detecting differences in phase of disease, with RS adjusted changes being second best. The GRC method accords most with actual clinical practice, in which health-care providers usually rely on patients' judgment if they are better, the same or worse. However, the question remains, which is the most meaningful and least biased outcome? The most sensitive outcome could be the most biased. If patients are aware that the phase of their disease is deteriorating, they may be more prone to assuming that their HRQOL must as a consequence be similarly declining, resulting in biased reports of GRC and possibly RS adjusted changes.

A possible explanation for the discrepancy between the pre-test and then-test assessment is the potential for recall bias. In HRQOL research, recall bias refers to memory distortion; that is if patients incorrectly recall their health condition at T1 [27,28]. However, in a study by Visser et al comparing different approaches to detect RS, recall bias did not invalidate then-test result [29]. A factor such as the length of period between measurements may affect the influence of recall bias. Like Visser and others [9,29], we used a relatively short interval between assessments (3 months). If we had chosen a shorter interval between pre-test and then-test, the patients could have remembered what they actually answered on the pre-test. A longer period between the initial measurement and the retrospective then-test would pose a considerable challenge to memory. The choice of 3 months in the present study was a compromise between these considerations. Another possible explanation for the observed results could be the "implicit theory of change". This theory suggests that patients begin with their presumed present state (post-test) and work backwards to their pre-test state (pre-test), and not on their perception of their health at a specific time point [27]. A consequence could be that patients view the decline in their HRQOL as bigger than it actually is because they believe their disease is progressing and that consequently their HRQOL must be deteriorating.

Although RS could be a challenge for the measurement and interpretation of self-reported HRQOL, adaption to illness could serve as a form of psychological buffer that helps reduce the stressful impact of a deteriorating health status. For most patients, living after being diagnosed with cancer is not the same as before. An important part of every cancer treatment is helping patients to adapt to their illness. Thus, the positive adaption we found in our study in patients saying that they deteriorated is actually a desired effect for the patients.

We chose to study MM patients because we anticipated large differences in HRQOL score between those who *improved *or *deteriorated*. A comparison with the results obtained with the EORTC QLQ-C30 in patients with other haematological diseases [30] and in solitaire cancers [31,32] indicates that patients' HRQOL is lower in MM than in several other malignant diseases.

The evaluation of external validity is important to enhance the transfer of results into the clinical routine. The strength of our study is that we included an almost representative sample of patients with MM within the South-Eastern Norway, although the median age was somewhat lower (66 years) than in a newly published population based study from Sweden (72 years) [33]. However, the mean EORTC QLQ-C30 scores for the whole sample in our study is comparable to a nationally representative study among MM patients in Denmark [30]. Given the representativeness of the patients included, we can expect the results to be relevant to other MM patients. We would also expect these findings to apply to other cancers or other illnesses, and we encourage confirmatory studies to investigate this.

## Conclusions

In our study, MIDs estimated from pre-test/post-test data appeared to be robust against RS in patients who *improved *over 3-months. This could indicate that RS has a minimal impact on the results in patients who respond to treatment, and that RS may not have an important impact on interpretation of changes reported in clinical trials where an improvement occurs. Although the ESs of the RSs were small, RS in *deteriorated *patients may augment MID estimates with up to 12 points and may have an important impact on the interpretation of changes reported in clinical trials.

## Competing interests

The authors declare that they have no competing interests.

## Authors' contributions

All authors conceived of the study, participated in the design of the study, performed the statistical analysis, read and approved the final manuscript. AKK performed the interviews of the patients.

## References

[B1] Howard GS Ralph KM Gulanick NA Maxwell SE Nance DW Gerber SK Internal Invalidity in Pretest-Posttest Self-Report Evaluations and a Re-evaluation of Retrospective Pretests Applied Psychological Measurement 1979 3 1 23 10.1177/014662167900300101

[B2] Sprangers MA Schwartz CE Integrating response shift into health-related quality of life research: a theoretical model Soc Sci Med 1999 48 1507 15 10.1016/S0277-9536(99)00045-3 10400253

[B3] Schwartz CE Sprangers MA Methodological approaches for assessing response shift in longitudinal health-related quality-of-life research Soc Sci Med 1999 48 1531 48 10.1016/S0277-9536(99)00047-7 10400255

[B4] Visser MR Smets EM Sprangers MA de Haes HJ How response shift may affect the measurement of change in fatigue J Pain Symptom Manage 2000 20 12 8 10.1016/S0885-3924(00)00148-2 10946164

[B5] Jansen SJ Stiggelbout AM Nooij MA Noordijk EM Kievit J Response shift in quality of life measurement in early-stage breast cancer patients undergoing radiotherapy Qual Life Res 2000 9 603 15 10.1023/A:1008928617014 11236851

[B6] Sharpe L Butow P Smith C McConnell D Clarke S Changes in quality of life in patients with advanced cancer: evidence of response shift and response restriction J Psychosom Res 2005 58 497 504 10.1016/j.jpsychores.2005.02.017 16125516

[B7] Hagedoorn M Sneeuw KC Aaronson NK Changes in physical functioning and quality of life in patients with cancer: response shift and relative evaluation of one's condition J Clin Epidemiol 2002 55 176 83 10.1016/S0895-4356(01)00438-3 11809356

[B8] Bernhard J Hurny C Maibach R Herrmann R Laffer U Quality of life as subjective experience: reframing of perception in patients with colon cancer undergoing radical resection with or without adjuvant chemotherapy. Swiss Group for Clinical Cancer Research (SAKK) Ann Oncol 1999 10 775 82 10.1023/A:1008311918967 10470423

[B9] Rees J Waldron D O'Boyle C Ewings P MacDonagh R Prospective vs retrospective assessment of lower urinary tract symptoms in patients with advanced prostate cancer: the effect of 'response shift' BJU Int 2003 92 703 6 10.1046/j.1464-410X.2003.04462.x 14616450

[B10] Andrykowski MA Donovan KA Jacobsen PB Magnitude and correlates of response shift in fatigue ratings in women undergoing adjuvant therapy for breast cancer J Pain Symptom Manage 2009 37 341 51 10.1016/j.jpainsymman.2008.03.015 18757176 PMC2682229

[B11] Schwartz CE Bode R Repucci N Becker J Sprangers MA Fayers PM The clinical significance of adaptation to changing health: a meta-analysis of response shift Qual Life Res 2006 15 1533 50 10.1007/s11136-006-0025-9 17031503

[B12] Kvam AK Fayers P Wisloff F What changes in health-related quality of life matter to multiple myeloma patients? A prospective study Eur J Haemato 2010 84 345 53 10.1111/j.1600-0609.2009.01404.x 20041946

[B13] Sirohi B Powles R Epidemiology and outcomes research for MGUS, myeloma and amyloidosis Eur J Cancer 2006 42 1671 83 10.1016/j.ejca.2006.01.065 16870424

[B14] Gulbrandsen N Hjermstad MJ Wisloff F Interpretation of quality of life scores in multiple myeloma by comparison with a reference population and assessment of the clinical importance of score differences Eur J Haematol 2004 72 172 80 10.1046/j.0902-4441.2003.00195.x 14962235

[B15] Jaeschke R Singer J Guyatt GH Measurement of health status. Ascertaining the minimal clinically important difference Control Clin Trials 1989 10 407 15 10.1016/0197-2456(89)90005-6 2691207

[B16] Aaronson NK Ahmedzai S Bergman B Bullinger M Cull A Duez NJ Filiberti A Flechtner H Fleishman SB de Haes JC The European Organization for Research and Treatment of Cancer QLQ-C30: a quality-of-life instrument for use in international clinical trials in oncology J Natl Cancer Inst 1993 85 365 76 10.1093/jnci/85.5.365 8433390

[B17] Fayers PM Aaronson NK Bjordal K Groenvold M Curran D Bottomley A on behalf of the EORTC Quality of life group The EORTC QLQ-C30 Scoring Manual 2001 3 European Organisation for Research and Treatment of Cancer, Brussels

[B18] Wisloff F Eika S Hippe E Hjorth M Holmberg E Kaasa S Palva I Westin J Measurement of health-related quality of life in multiple myeloma. Nordic Myeloma Study Group Br J Haematol 1996 92 604 13 10.1046/j.1365-2141.1996.352889.x 8616024

[B19] Cohen J Statistical power analysis for the behavioral sciences 1988 Hillsdale, N. J.: Laurence Erlbaum

[B20] Fayers PM Machin D Quality of life: the assessment, analysis, and interpretation of patient-reported outcomes 2007 Chichester: J. Wiley

[B21] Sprangers MA Van Dam FS Broersen J Lodder L Wever L Visser MR Oosterveld P Smets EM Revealing response shift in longitudinal research on fatigue--the use of the thentest approach Acta Oncol 1999 38 709 18 10.1080/028418699432824 10522761

[B22] Gulbrandsen N Wisloff F Brinch L Carlson K Dahl IM Gimsing P Hippe E Hjorth M Knudsen LM Lamvik J Lenhoff S Lofvenberg E Nesthus I Nielsen JL Turesson I Westin J Health-related quality of life in multiple myeloma patients receiving high-dose chemotherapy with autologous blood stem-cell support Med Oncol 2001 18 65 77 10.1385/MO:18:1:65 11778972

[B23] Dubois D Dhawan R van d V Esseltine D Gupta S Viala M de la Loge C Descriptive and prognostic value of patient-reported outcomes: the bortezomib experience in relapsed and refractory multiple myeloma J Clin Oncol 2006 24 976 82 10.1200/JCO.2005.04.0824 16432077

[B24] Ring L Hofer S Heuston F Harris D O'Boyle CA Response shift masks the treatment impact on patient reported outcomes (PROs): the example of individual quality of life in edentulous patients Health Qual Life Outcomes 2005 3 55 62 10.1186/1477-7525-3-55 16146573 PMC1236951

[B25] Razmjou H Schwartz CE Yee A Finkelstein JA Traditional assessment of health outcome following total knee arthroplasty was confounded by response shift phenomenon J Clin Epidemiol 2009 62 91 6 10.1016/j.jclinepi.2008.08.004 19095168

[B26] Vogelzang NJ Breitbart W Cella D Curt GA Groopman JE Horning SJ Itri LM Johnson DH Scherr SL Portenoy RK Patient, caregiver, and oncologist perceptions of cancer-related fatigue: results of a tripart assessment survey. The Fatigue Coalition Seminars in Hematology 1997 34 3 Suppl 2 4 12 9253778

[B27] Norman G Hi! How are you? Response shift, implicit theories and differing epistemologies Qual Life Res 2003 12 239 49 10.1023/A:1023211129926 12769136

[B28] Fayers PM Hays RD Assessing quality of life in clinical trials: methods and practice 2005 Oxford: Oxford University Press

[B29] Visser MR Oort FJ Sprangers MA Methods to detect response shift in quality of life data: a convergent validity study Qual Life Res 2005 14 629 39 10.1007/s11136-004-2577-x 16022057

[B30] Johnsen AT Tholstrup D Petersen MA Pedersen L Groenvold M Health related quality of life in a nationally representative sample of haematological patients Eur J Haematol 2009 83 139 48 10.1111/j.1600-0609.2009.01250.x 19284418 PMC2730555

[B31] Bottomley A Therasse P Piccart M Efficace F Coens C Gotay C Welnicka-Jaskiewicz M Mauriac L Dyczka J Cufer T Lichinitser MR Schornagel JH Bonnefoi H Shepherd L Health-related quality of life in survivors of locally advanced breast cancer: an international randomised controlled phase III trial Lancet Oncol 2005 6 287 94 10.1016/S1470-2045(05)70100-5 15863376

[B32] Zenger M Hinz A Stolzenburg JU Rabenalt R Schwalenberg T Schwarz R Health-related quality of life of prostate cancer patients compared to the general German population: age-specific results Urol Int 2009 83 166 70 10.1159/000230018 19752611

[B33] Turesson I Velez R Kristinsson SY Landgren O Patterns of Improved Survival in Patients With Multiple Myeloma in the Twenty-First Century: A Population-Based Study J Clin Oncol 2010 28 830 4 10.1200/JCO.2009.25.4177 20038719 PMC2834396

